# “In Weapons We Trust?” Four-culture analysis of factors associated with weapon tolerance in young males

**DOI:** 10.1371/journal.pone.0317182

**Published:** 2025-03-20

**Authors:** Marek Palace, Brandon May, Neil Shortland, William Brown, David Mcllroy, Manish Madan, Anna Bokszczanin, Dominika Gurbisz, Sarah Daly, Laura Hansen, Rakhi Tripathi, Divyashree Harjai, Sukdeo Ingale, Olga Dussart, Wenping Jiang, Vie Palle

**Affiliations:** 1 School of Psychology, Liverpool John Moores University, Liverpool, United Kingdom; 2 School of Psychology, Florida Institute of Technology, Melbourne, Florida, United States of America; 3 Center for Terrorism and Security Studies, University of Massachusetts Lowell, Lowell, Massachusetts, United States of America; 4 School of Psychology, University of Bedfordshire, Luton, United Kingdom; 5 School of Social and Behavioral Sciences, Stockton University, Galloway, New Jersey, United States of America; 6 Institute of Psychology, Opole University, Opole, Poland; 7 Doctoral School of Social Sciences, Institute of Psychology, Jagiellonian University, Kraków, Poland; 8 Criminal Justice Department, St Vincent College, Latrobe, Pennsylvania, United States of America; 9 College of Art and Sciences, Western New England University, Springfield, Massachusetts, United States of America; 10 Department of Information Technology, Fore School of Management, New Delhi, India; 11 School of Criminal Law and Military Law, Raksha Shakti University, Lavad, India; 12 Department of Law, Vishwakarma University, Pune, India; 13 Institute of Psychology, Polish Academy of Sciences, Warsaw, Poland; 14 Centre for Doctoral Training in Distributed Algorithms, University of Liverpool, Liverpool, United Kingdom; 15 School of Arts, Languages and Culture, University of Manchester, Manchester, United Kingdom; University of Padova, ITALY

## Abstract

Addressing the under-researched issue of weapon tolerance, the paper examines factors behind male knife and gun tolerance across four different cultures, seeking to rank them in terms of predictive power and shed light on relations between them. To this end, four regression and structural equation modelling analyses were conducted using samples from the US (*n* = 189), India (*n* = 196), England (*n* = 107) and Poland (*n* = 375). Each sample of male participants indicated their standing on several dimensions (i.e., predictors) derived from theory and related research (i.e., *Psychoticism, Need for Respect, Aggressive Masculinity, Belief in Social Mobility and Doubt in Authority*). All four regression models were statistically significant. The knife tolerance predictors were: *Aggressive Masculinity* (positive) in the US, Poland and England, *Belief in Social Mobility* (negative) in the US and England, *Need for Respect* (positive) in India and *Psychoticism* (positive) in Poland. The gun tolerance predictors were: *Psychoticism* (positive) in the US, India and Poland, *Aggressive Masculinity* (positive) in the US, England and Poland, and *Belief in in Social Mobility* (negative) in the US, *Belief in Social Mobility* (positive) and *Doubt in Authority* (negative) in Poland. The Structural Equation Weapon Tolerance Model (WTM) suggested an indirect effect for the latent factor *Perceived Social Ecological Constraints* via its positive relation with the latent factor *Saving Face*, both knife and gun tolerance were predicted by *Psychoticism*.

Although violence in human cultures is typically seen as normal [1], the factors associated with its inception and expression can vary widely, likely shaping attitudes towards carrying weapons, such as guns and knives, which are the focus of this paper. Some of the main factors argued to influence violence include: poverty and inequality [2], levels of disadvantage in the community [3], socio-geo-cultural elements associated with religion [4], collective memory of intergroup conflict [5], scepticism about the rule of law [6] and perceived threat [7]. Whilst the concept of culture has been often associated with values seen as worthy of defending [8], some newer approaches suggest viewing culture as a repertoire of strategies for negotiating social situations [7,9–11]. Thus, in the current paper, we adapt this more recent approach.

Acknowledging that violence is dependent on context and shaped by culturally-determined factors [12], we respond to the need for the search of aggression patterns across different nations suggested by Archer [13]. Specifically, we address a gap in comparative research on young males in the US, England, India and Poland, which are respectively associated with individualistic, collectivist and mixed cultural values and different attitudes to violence and weapons [14]. The choice of the first three cultures was driven by the common language – the three most populated English-speaking nations. The choice of Poland, a non-anglophile country, was driven by the links that it has with the first three, like collectivist cultural elements - similarity to India - increasing individualism and relatively unregulated economy - similarity to the US and England. Mindful of the research bias towards mono-culturalism, which might be partly down to inherent methodological issues in cross-cultural research [15, 16], we focus on the underexplored theme of weapon tolerance.

## The American context

American history books are replete with narratives from the *Wild West*, with larger-than-life characters, like *Billy the Kid*. Hollywood films glorified the unruly, untamed western states in most Westerns, most notably made popular by actors John Wayne and Clint Eastwood. Historically, the Americans’ relationship with guns have been politically nuanced. While US gun culture has association with hunting motifs, now it is self-protection that is argued to play a central role [17]. In the regard, Conservative figures present them as tools for keeping their family members safe from threats posed by strangers [18]. While glamorisation has arguably been driven by Hollywood depictions – and the National Rifles Association mythologising weapon-carrying as a symbol of respect, status and courage [17] - commercials romanticise it in terms of affirming safety, freedom and self-reliance [18].

Approximately 30% of American adults report owning a gun, with one-in-ten of US adults living in households where there are guns [19]. How many illegal guns remain in circulation is unknown as it is part of the hidden, *dark* numbers missing from crime statistics. That being said, America has more guns than people [20] reflecting a cultural context in which figures such as Old West sheriffs or lawmen are often depicted in a romanticised or aspirational manner. Indeed, this cultural ethos extends to vigilantism, which, under the constitutional framework of *citizen’s arrest*, appears to endorse the idea of individuals taking the law into their own hands.

The federal law has no restriction on openly carrying a firearm, except for rules that apply to any property that is owned or operated by the federal government [21]. When there have been school shootings, there have even been suggestions that teachers should be armed in the classroom. While most states have no restrictions on knife-carrying in general, certain types and knives (e.g., switchblades) are illegal in some states. While the most common weapon used to commit homicide in the US remain firearms, they are followed by knives that are used to kill, on average, more than 1500 people each year [22].

## The Indian context

India is renowned for having some of the strictest gun laws in the world. While citizens are legally allowed to own and carry firearms, this privilege is not constitutionally guaranteed, and obtaining a gun license is challenging. The application process is lengthy and complex, often requiring legal assistance and numerous documents, such as medical and police certificates. In contrast to firearms, however, India has a rich socio-historical connection to bladed weapons, rooted in one of its greatest material contributions: *Deccani wootz steel*. Often celebrated as the *wonder material of the Orient* [23], this steel was used to craft the famed *Damascus blades*. These high-quality weapons were not only functional but also artistically adorned with carvings and inlays of brass, silver, and gold [24], embedding swords and knives into Indian culture as both tools and symbols of heritage.

In comparison to the United States, India has a lower homicide rate, with the United Nations Office on Drugs and Crime reporting 3.22 per 100,000 in 2020 compared to 5.35 in the U.S. However, India’s approach to crime data collection differs significantly. The National Crime Records Bureau (NCRB), a state agency, does not track knife crime specifically, and its crime categorisation diverges from that of the U.S., England, or other Western nations. Furthermore, India lacks independent, non-governmental institutions dedicated to gathering comprehensive crime statistics. As a result, there is no official data on knife crime, its causes, or its impact, nor on strategies for its prevention and reduction.

## The English context

After 2005, a significant rise in knife crimes was recorded in European countries [25]. According to the Scottish Crime and Justice Survey, in 2013-14 the knife was the most commonly used weapon (39%), and sharp objects were the most commonly used instruments in homicide in Scotland. Since 2009, over £2.5 million were spent on the “*No Knives, Better Lives*” initiative [26] that started with the intention to educate young people about the dangers and consequences of knife-carrying. Glasgow’s Violence Reduction Unit (VRU), established in 2005, has also played a significant role by encouraging local firms to hire former offenders and offering mentorship services to jobseekers. Since then, the homicide rate in Scotland has dropped by 60% [27].

In England, knife attacks have recently surged to unprecedented levels. In the year ending March 2018, 285 people were killed in knife or sharp instrument-related homicides, marking the highest number since records began in 1946. During the same period, approximately 40,000 offenses involving a knife or sharp object were recorded in England and Wales, an 8% increase from the previous year, according to the Office for National Statistics (ONS). Meanwhile, firearm crime levels in the UK remain among the lowest globally, a fact widely attributed to the country’s stringent gun ownership laws. However, the UK is not entirely free of firearm offences. According to the National Crime Agency, 5,750 firearm-related offenses were reported in England and Wales in the year ending March 2022. Despite these figures, the UK’s overall homicide rate remains low, with the United Nations Office on Drugs and Crime reporting a rate of just 1.20 per 100,000 people in 2020.

## The Polish context

Poland transitioned from the Soviet-imposed communist system to a democratic free-market system in 1989, joining the North Atlantic Treaty Organization (NATO) in 1999 and the EU in 2004. The transition was marked by a rapid increase in violent crimes oftentimes committed with smuggled guns, knives and baseball bats, which was attributed to less strict border checks, more limited police powers and a legal system that became more liberal [28]. The legal and freely available baseball bat became associated with tracksuit-clad soccer hooligans and extortion gangs demanding protection fees from new private businesses previously banned under communism [29] – a point that remains relevant to research on weapon-carrying in Poland in socio-historical terms.

Poland’s participation in the Schengen Zone (where most European countries, abolished their internal borders, for the free and unrestricted movement of people), coupled with the key drug transit route from Asia to Western Europe, facilitate the flow of illegal firearms that are mostly held by organised criminal groups. Despite this, and notwithstanding some of the most liberal laws regarding bladed instruments, Poland’s homicide rate in 2020 was half of that of the UK: 0.67 per 100,000. The following Table 1 illustrates the four contexts in terms of key statistics:

**Table 1:** Weapons, Violence and Country [30].

Factor	US	UK	India	Poland
Human development index	.927	.940	.644	.881
GDP per capita in $	85,373	58,880	10,123	49,060
% of world gun deaths	14.85	.006	5.9	.004
Stabbing mortality rate	.60	.08	.64	.49
Homicide rate per 100,000	4.96	1.20	3.08	.73
Violence rate per 100,000	3.96	.004	.57	.009
Guns per 100 people	120.50	5.10	5.30	2.50

Despite some obvious similarities between the individualist cultures of the United States and United Kingdom [31], the attitudes to weapons remain vastly different [32], the most popular items being respectively guns [32] and knives [33,34]. Whereas the former are constitutional – for instance under the US 2nd amendment - the latter remain illegal to carry in public in the UK, where even a screwdriver may hold criminal culpability without reasonable plausibility. Largely collectivist India and (to a lesser extent) Poland (generally conservative Catholic community with increasing individual aspirations) both prohibit personal possession of firearms without special licenses that are not practically available to most citizens.

## Current research

Notwithstanding the differences in weapon prevalence (e.g., guns vs. knives) between countries, adolescent weapon-carrying is widely regarded as a global issue [35]. One partial explanation for weapon-carrying tolerance is offered by *Protection Motivation Theory* [36], which covers how people perceive or evaluate any risk and how they adopt protecting behaviours or measures. The theory suggests that four cognitions facilitate motivations for self-defence: *risk severity, risk vulnerability, self-efficacy at reducing risk*, and the *response efficacy of the advocated behaviour*. The theory also proposes that such motivations can be compromised by the apparent costs of risk-reduction and likely benefits of risk-increasing behaviour (e.g., weapon-carrying). The involved processes thus include threat appraisal (i.e., severity, vulnerability, and benefits) and coping appraisal (i.e., self-efficacy, response efficacy, and costs).

Despite a wealth of research on weapon-carrying [33,35], it is unclear what factors lie behind attitudes towards its tolerance, which may not necessarily imply acceptance of violence. Our present paper aims to address this gap by building upon a structural equation *Knife Tolerance Model* (KTM) by Palasinski et al., [33], which covers factors associated with knife-carrying tolerance in England. KTM was partially informed by a systematic review and meta-analysis of cross-sectional and longitudinal research on weapon-carrying [37] and grounded in terms of self-protection (construed as: Physical Defence Ability and Need for Respect). The KTM revealed significant intercorrelations between physical defence ability, limited trust in authority (e.g., in the police), limited control over one’s status and the need for respect (i.e., predictor factors), and how they predict aggressive masculinity (i.e., ‘macho’ culture). Importantly, the KTM also identified two significant underlying (i.e., latent and not immediately apparent) factors: perceived social ecological constraints (i.e., socioeconomic limitations, like deprivation and few opportunities) and saving face (i.e., honor and inter-male competition).

Identifying the complex processes underlying weapon carrying tolerance has both theoretical and practical implications, especially when we seek to develop evidence-based intervention campaigns. Thus, in the present paper, we answer the call for more in-depth research on weapon-carrying [38] and examine the validity of KTM concepts with regards to both knives and guns, as well as their relevance to different cultures.

**Fig 1:**
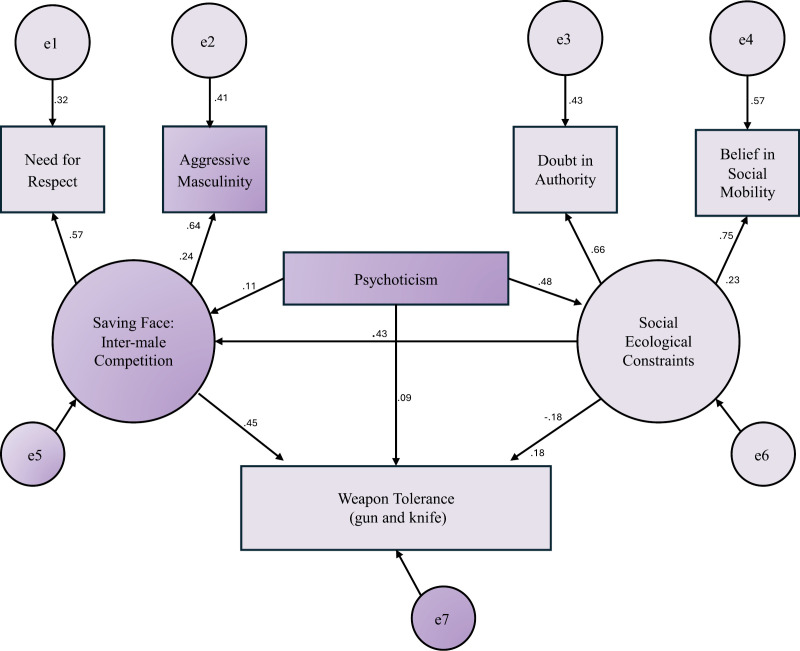
Structural Equation Weapon Tolerance Model.

Based on the KTM [33], we hypothesized that our proposed Structural Equation Weapon Tolerance Model (Fig 1; featuring the same main scales as KTM) would also be statistically significant with regards to the two weapons (i.e., guns and knives) and across different cultures. Given the recent research implying some limitations of *Protection Motivation Theory* [36], and the absence of personality factors in KTM, we also included the dimension of psychoticism in WTM,

Since it is mostly men who engage in physical violence [39] in real life-settings [40] across different cultures [13], and who were experimentally shown to find guns and knives faster than women [41], in the present research all our participants are male. To highlight the distinct differences on the cross-cultural spectrum, reflect separate data collection time windows and to aid readability, the research is presented in the form of four samples. As there is enormous cultural variance in each examined country and our samples are regional, the identified differences should be treated more like an introduction to research on cultural aspects of weapon-carrying whose fuller tapestry should be pursued in further studies. Following the Institutional Review Board, all participants confirmed their written consent on the introductory study page.

## Methodology

The anonymous survey study, which involved the same questions and scale items for each country (all presented in English except for the Polish sample faced with a professionally translated Polish version) was introduced to all participants as ‘aspects of aggression’. Participants were recruited via opportunity sampling; it took approximately 10 minutes to complete the survey featuring a number of 5-point anchored Likert-type scales that were presented to participants without any labels.

The dependent factors (and their respective 5-item scales were kept deliberately brief to encourage completion rates) were derived from and based on the key concepts from closely related papers on violence and knives [33,34]. Thus, the dependent factors included: Knife Tolerance (e.g., The mass media exaggerate the dangers of carrying a blade; α=.73) and Gun Tolerance (e.g., The mass media exaggerate the dangers of gun-carrying; α=.71).

The key independent factors were based on the same respective 5-item scales used in the KTM paper [33]. They included: Need for Respect (e.g., Being respected by others is important; Cronbach’s α=.77), Aggressive Masculinity (e.g., Controlled violence can be an asset; α=.73), Belief in Social Mobility (e.g., There are opportunities available; α=.79), Doubt in Authority (e.g., The authorities are out of touch; α=.81). To aid readability for those unfamiliar with KTM, the factors were phrased slightly differently in the WTP that also features Psychoticism (e.g., Most people cannot be trusted; α=.68). The internal reliability levels were based on response from all participants.

A total of *N* = 189 (predominantly White and US-born) male participants’ residing in the (North Central) US took full part in the study (*M*_*age*_ = 22.12, *SD*_*age*_ = 8.41). They came from diverse socio-economic (mostly locally defined as lower middle income) family backgrounds and were recruited online (via the university study recruitment system and Facebook by posting the survey link on sites oriented towards male interests).

A total of *N* = 196 male participants’ resident in India took full part (*M*_*age*_ =  21.20, *SD*_*age*_ = 7.68). They shared the same Hindu ethnicity but came from different socio-economic (mostly locally defined as lower middle income and India-born) family backgrounds and were recruited on a college campus in the city of Pune in the western peninsular state of Maharshtra dominated by Hindus. Given the local restrictions on online studies, a paper version of the survey was administered.

A total of *N* = 107 male (predominantly White and UK-born) participants’ resident in (Northwestern) England took full part (*M*_*age*_ = 23.27, *SD*_*age*_ = 97). They came from different socio-economic (mostly locally defined as lower middle income) family backgrounds and were recruited online (via the university study recruitment system and Facebook by posting the survey link on sites oriented towards male interests).

A total of *N* = 375 male (White and Poland-born) participants’ resident in Southwestern Poland took full part (*M*_*age*_ = 21.00, *SD*_*age*_ = 6.99). Given Poland’s proverbial cultural and ethnic homogeneity [42], in this sample we purposefully included young men without official violent record (*n* = 156; of locally defined lower middle income family backgrounds (recruited via the university study recruitment system) and those convicted of violent offences (*n* = 219; of locally defined low-income family backgrounds who completed the paper version of the survey). This, in turn, will partially reflect some of the diversity featuring in the other three ethnically mixed samples. As such stratification in the Polish sample is skewed towards violent offenders, who are more likely to carry knives [33] and guns [32], its use will also allow us to see if (and how) the mixed Polish sample will differ from the other three samples in terms of regression results. All participants were assured of anonymity and confidentiality, and no personally identifiable information was collected. The uploaded data are also available by contacting the first author.

## Results

### The US sample

The total variance explained by the knife model as a whole was 24.8%, *R*^*2*^ = .248, *F* (5, 189) = 13.452, *p* < .001. The only significant positive predictor was Aggressive Masculinity (*β*=.406, *p* < .001). The only significant negative predictor was Belief in Social Mobility (*β*=-.162, *p* = .030). The total variance explained by the gun model as a whole was 32%, *R*^*2*^ = .324, *F* (5, 189) = 19.157, *p* < .001. The only significant positive predictors were Aggressive Masculinity (*β*=.407, *p* < .001) and Psychoticism (*β*=.184, *p* = .007). The only significant negative predictor was Belief in Social Mobility (*β*=-.226, *p* = .002).

### The Indian sample

The total variance explained by the knife model was 5.7%, *R*^*2*^ = .057, *F* (5, 196) = 3.38, *p* = .006. The only significant positive predictor was Need for Respect (*β*=.24, *p* = .035). The total variance explained by the gun model was 2%, *R*^*2*^ = .020 F (5, 196) = 1.789, *p* = .117. The only significant positive predictor was Psychoticism (*β*=.019, *p* = .05).

### The English sample

The total variance explained by the knife model was 36.6%, *R*^*2*^ = .356, *F*(5, 107) = 12.807, *p* < .001. The only significant positive predictor was Aggressive Masculinity (*β*=.514, *p* < .001). The only significant negative predictor was Belief in Social Mobility (*β*=-.332, *p* = .001). The total variance explained by the gun model was 39%, *R*^*2*^ = .392, *F*(5, 107) = 14.796, *p* < .001. The only significant positive predictor was Aggressive Masculinity (*β*=.488, *p* < .001).

### The Polish sample

The total variance explained by the knife model was 15.5%, *R*^*2*^ = .155, *F* (5, 375) = 14.71, *p* < .001. The only significant positive predictors were Aggressive Masculinity (*β*=.275, *p* = .001) and Psychoticism (*β*=.196, p = .001). The total variance explained by the gun model was 13.4%, *R*^*2*^ = .134, *F* (5, 375) = 12.655, *p* < .001. The only significant positive predictors were Aggressive Masculinity (*β*=.291, *p* = .001), Psychoticism (*β*=.145, *p* = .008) and Belief in Social Mobility (*β*=.112, *p* = .049). The only significant negative predictor was Doubt in Authority (*β*=-.132, *p* = .024). The differences between Polish participants with and without violent conviction were not found to be significant.

### Multicultural analyses

First, we examined baseline culture differences between the factors before developing inferential multiple regression and structural equation models. We used this to test how well the variables comprising the two latent factors ‘Saving Face’ (based on Need for Respect and Aggressive Masculinity) and ‘Social Ecological Constraints’ (based on Belief in Social Mobility and Doubt in Authority) would vary by culture. Weapon tolerance (i.e., knife or gun) was regressed on Psychoticism, Need for Respect, Aggressive Masculinity, Belief in Social Mobility and Doubt in Authority.

### Baseline culture differences

Using a one-way ANOVA with a four-level categorical Culture variable (US, India, England and Poland) and gun tolerance as dependent variable, shows significant differences between the cultures *F*(3, 875) = 25.748, *p* < .001. More specifically, post-hoc Tukey tests show that Indian participants (*M* = 4.30, *SD* = 1.13) had higher gun tolerance than Poland’s (*M* = 3.32; *SD* = 1.51), US (*M* = 3.26; *SD* = 1.71), and England’s (*M* = 3.02; *SD* = 1.72) participants, *p* < .001. The differences between the gun tolerance of Poland’s and England’s participants, and Poland’s and US participants were not significant, respectively, *p* = .265 and *p* = .968. The difference between the gun tolerance of England and US-based participants was not significant, *p* = .557.

In terms of knife tolerance, the ANOVA shows significant differences between the cultures, *F* (3, 875) = 50.244, *p* < .001. India’s participants (*M* = 4.47, *SD* = 1.11) had more knife tolerance than Poland’s (*M* = 3.45, *SD* = 1.35), US (*M* = 3.03; *SD* = 1.44) and England’s (*M* = 2.86, *SD* = 1.52) participants, *p* < .001. The differences between the knife tolerance of Poland’s and England’s participants, and Poland’s and US participants were both significant, respectively *p* < .001 and *p* = .003. The difference between the knife tolerance of England and US-based participants was not significant, *p* = .73.

### Multicultural weapon tolerance multiple regression models

The Knife and Gun Tolerance regression models were statistically significant: Knife Tolerance Model *F*(5, 843) =  21.11, *p* < .001; Gun Tolerance Model *F*(5, 844) =  21.50, *p* < .001. The left-hand and right-hand panels of Table 2 display the standardized and unstandardized beta coefficients for predictors in the Knife and Gun Tolerance models. As indicated, both models accounted for 11% of the variance in weapon tolerance. As predicted, the Saving Face variables (i.e., Need for Respect and Aggressive Masculinity) accounted for a significant proportion of the variance in Weapon Tolerance over and above Psychoticism or Social Ecological variables (i.e., Belief in Social Mobility or Doubt in Authority). Table 2 features the multiple regression results for the entire sample (US, India, England and Poland). Table 3 features the related descriptive statistics.

**Table 2:** Raw and standardized coefficients from a standard regression in which weapon tolerance (knife vs. gun) was regressed for each culture on: psychoticism, need for respect, aggressive masculinity, belief in social mobility, and doubt in authority.

Variable	Knife Tolerance				Gun Tolerance			
	*F*	B	SE B	*β*	*F*	*B*	*SE B*	*β*
**Four Culture Model** ^†^								
**(N = 867)**								
Psychoticism	21.11^**^	.08	.04	.08^*^	21.50^**^	.08	.08	.09^*^
Need for Respect		.77	.04	.17^**^		.19	.04	.19^**^
Aggressive Masculinity		.21	.04	.20^**^		.19	.04	.19^**^
Belief in Social Mobility		−.05	.04	.05		.01	.04	.00
Doubt in Authority		.00	.04	.00		−.04	.04	.00^*^
Constant		−.00	.03	–		−.01	−.01	–
**USA (N = 189)**								
Psychoticism	13.45^**^	.18	.10	.13	19.16^**^	.29	.11	.18^**^
Need for Respect		.05	.08	.05		.12	.09	.09
Aggressive Masculinity		.38	.07	.41^**^		.46	.08	.41^**^
Belief in Social Mobility		−.16	.07	−.16^*^		−.27	.08	−.23^**^
Doubt in Authority		−.10	.07	−.11		−.12	.08	−.11
Constant		2.23	.69	–		1.79	.79	–
**England (N = 107)**								
Psychoticism	12.81^**^	.13	.12	.10	14.80^**^	.19	.13	.13
Need for Respect		.11	.10	.10		.20	.11	.15
Aggressive Masculinity		.51	.09	.51^**^		.56	.10	.49^**^
Belief in Social Mobility		−.37	.10	−.33^**^		−.51	.12	−.40^**^
Doubt in Authority		−.02	.10	−.02		.02	.12	.02
Constant		2.19	.83	–		1.81	.92	–
**Poland (N = 375)**								
Psychoticism	14.71^**^	.20	.05	.20^**^	12.66^**^	.16	.06	.15^**^
Need for Respect		.01	.05	.01		.01	.06	.01
Aggressive Masculinity		.26	.06	.30^**^		.30	.06	.29^**^
Belief in Social Mobility		.02	.06	.02		.14	.07	.11^*^
Doubt in Authority		.01	.05	.01		−.13	.06	−.13^*^
Constant		1.35	.30	–		1.31	.34	–
**India (N = 196)**								
Psychoticism	3.38^**^	−.05	.04	−.08	1.79	.11	.05	.17^*^
Need for Respect		.23	.08	.24^**^		−.01	.08	−.01
Aggressive Masculinity		.02	.08	.01		.01	.06	.01
Belief in Social Mobility		.02	.03	.02		.03	.04	.06
Doubt in Authority		.30	.09	.07		.07	.06	.09
Constant		3. 90	.65	–		4.75	.69	–

*Notes:*

† Due to statistically significant scale invariance violations between cultures, the data were converted to z-scores for the four culture analyses.

* p < .05;

** p < .01. Multicultural Knife Tolerance Model: (adjusted R^2^ = .11, p < .001); Multicultural Gun Tolerance Model: (adjusted R^2^ = .11, p < .001).

**Table 3:** Weapon tolerance descriptive statistics.

Factor	MD	SD
Knife Tolerance US	3.03	1.44
Gun Tolerance US	3.26	1.71
Knife Tolerance India	4.47	1.11
Gun Tolerance India	4.30	1.13
Knife Tolerance England	2.86	1.52
Gun Tolerance England	3.02	1.72
Knife Tolerance Poland	3.45	1.35
Gun Tolerance Poland	3.32	1.51

### Structural equation weapon tolerance model (WTM)

The significant Chi Square value of our WTM may be interpreted as a limitation (*χ*^*2*^ = 6.01, df = 5, *p* < .05) in terms of model fitness. However, the Chi Square value is deemed to be oversensitive to model rejection especially with larger samples and the χ2/df ratio = 1.20. Note, this is taken as a more reliable index when under 3 as in this case). Nevertheless, the focus was put on the root mean square error of approximation (RMSEA = .01), comparative fitness index (CFI = .90), values and Akaike information criteria [AIC] = 50.01. Acknowledging that SEM indices do not have absolute cut-off point [43], the values indicate a satisfactory (albeit imperfect) fit, particularly if Likert scales are used and if the model is interpreted with caution [44]. The model (Fig 1) is also supported by high factor loadings (.57,.64,.66 &.75) and the moderate parameter estimate between the two latent variables (*β*=.43).

The model found low to high correlations (.09 to.75) between intercept and slope factors. All direct path correlations were statistically significant (*p* < .05). As anticipated, the latent factor, Saving Face (*β*= 45) directly predicted Weapon Tolerance (*β*=.43). Unexpectedly Perceived Social Ecological Constraints (PSEC) had a negative effect on Weapon Tolerance (direct standardized coefficient: *β*=-.18). While Psychoticism had more effect on Perceived Social Ecological Constraints (standardized coefficient: *β*=.48) than on Saving Face (standardized coefficient: *β*=.11), the former (PSEC) predicted Doubt in Authority (standardized coefficient: *β*=.66).and Belief in Social Mobility (*β*=.75). The latter (Saving Face), on the other hand, predicted Need for Respect (*β*=.57) and Aggressive Masculinity (*β*=.64).

There is an indirect effect (*p* < .001) from Psychoticism through Social Ecological Constraints to Saving Face (respectively:.48 and.43). These are multiplied to obtain the indirect effect that becomes (0.21). This combination accounts for an *R*^*2*^ = .24 on Saving Face (i.e., 24%,), meaning a substantial amount of variance is explained.

In addition, there are two other mediated relationships within the model. The direct effect from Psychoticism to Weapon Tolerance (.09) is mediated by its indirect routes through Social Ecological Constraints and Saving Face. The indirect route through Social Ecological Constraints is.48 x -.18 (-.09), and the indirect route through Saving Face is.11 x.45 (.05). These combined explain 18% of the variance on Weapon Tolerance (*R*^*2*^ = .18). The model therefore accounts for a non-trivial level of variance.

## Sample-specific discussion

### The US sample

In the US, both knife and gun models turned out to be statistically significant, thus lending credence to the original British knife-tolerance model [18]. The positive predictor role of Aggressive Masculinity and negative predictor role of Belief in Social Mobility applied to both knives and guns, suggesting little distinction between the tolerance of the two weapons. Given the rising social inequality, both Aggressive Masculinity and Belief in Social Mobility make sense in the US context. Here, a shared psychological process seems to underline both knife and gun tolerance, questioning the link between a specific ‘gun culture’, vs. a generalist ‘weapons culture’.

### The Indian sample

In India, only the knife model was statistically significant, which corresponds to the Indian tradition of swords dating back to the antiquity [45], as well as to the current Indian legislation that heavily restricts civilian possession of firearms. The positive predictor role of Need for Respect can be understood in the traditional Indian values that emphasize honor, respect and family name [46]. The India results are also consistent with the previous work on knife-carrying tolerance [33], specifically, the structural equation Knife Tolerance Model showing a positive correlation between Need for Respect and knife-carrying acceptance in England. Thus, saving face (i.e., honor) may be cross-culturally important in human interpersonal violence [8,46].

### The English sample

Like in the case of US results, both knife and gun models were statistically significant in England, which may be reflected by the relative cultural similarity of TV shows, movies and music. Such cultural similarity can also help explain the positive predictor role of Aggressive Masculinity and low British social mobility [47]. This lends weight to the socio-cultural similarities with the US, including generally conservative individualistic values and media portrayals of masculinity in terms strength and power [48].

### The Polish sample

Both knife and gun models were statistically significant, echoing their joined relevance found in both the US and English samples. The resemblance can also be traced to the positive role of Aggressive Masculinity, which probably reflects the heavy presence of convicted violent offenders in the sample. The roles of Psychoticism and Doubt in Authority, however, appear to be more complex and may warrant a separate follow-up study that is likely to be more illuminating than speculation without additional data. Future work in Poland should attempt to replicate the culture-specific associations to determine if it is part of a systemic self-report bias, repressive coping [49], or sampling variation. As the differences between Polish participants with and without violent conviction were not found to be significant, this may be down to our inclusion of relatively minor violent offences (which were far more common than the more serious ones involving grievous bodily harm).

In the next sections, we discuss our multicultural findings, i.e., multiple regression and structural equation models, covering the comparison between KTM and WTM.0872490.

## General discussion

The main purpose of this paper was to address the deficit in cross-cultural research on factors associated with tolerance of knives and guns across different cultures. Overall, the results support the cross-cultural relevance of the KTM, presenting a new structural equation Weapon Tolerance Model whose constructs go beyond the limited Protection Motivation Theory and beyond a simple KTM replication study. While Aggressive Masculinity and Need for Respect showed cross-cultural importance, some other factors (such as Belief in Social Mobility) showed cultural, and also weapon-specific effects. More specifically, in case of the US, English and Polish samples, both knife and gun models were statistically significant (in case of the Indian sample, only the knife model was significant).

While the exact reasons for this difference are unclear, it is likely down to the very distinctive Indian culture, which compared to the other three cultures is much older and whose tradition is steeped in ornate bladed weapons [23,24] rather than firearms. Avoiding speculation unwarranted by data, disambiguating this difference would likely require a separate study involving a broader range of psychological and cultural factors, which might also be partially informed by an additional qualitative study. Thus, such a culture (potentially coupled with other unexamined factors) likely played a bigger role in the resulting difference than the stratified nature of the Polish sample featuring violent offenders and non-offenders.

Despite some idiosyncrasies (like the positive predictor of Need for Respect in the Indian sample and other predictors in the Polish sample), an overlap of certain predictor factors was found, the positive one being Aggressive Masculinity (US, England, Poland) and negative one being Belief in Social Mobility (US, England, Poland).

We cautiously speculate that the lack of significant differences in reported gun tolerance between US, England and Poland-based participants, along with the lack of significant differences in knife tolerance between US and England-based participants might be down to the widespread cultural influence of violence-glamorising mass media [48] dominant in the three cultures. In such productions, the main *good guy* underdog protagonists are generally less violent than their usually better-armed adversaries. Such influence contrasts sharply with India’s post-colonial Bollywood themes of less graphic but more justified violence [50,51] that have been already associated with contributing towards juvenile delinquency [52], which might potentially shed some, but limited, light on Indian participants’ higher levels of gun and knife tolerance.

Although the four samples come from separate countries that represent distinct regional cultures, the data were collected in selected places that may not fully represent the nations’ attitudes and fully capture their diverse elements. Given the role of socialisation environment [53], we argue that the samples used are more representative of the specific national regions rather than the four countries at large, meaning that the models based on different samples from the American ‘Bible Belt’, Northern states of India (such as Muslim-dominated Uttar Pradesh, West Bengal and Bihar) and England’s most ethnically diverse areas (Luton, Slough and Newham) would likely result in different models shaped by their unique cultural values. Although the used convenience sampling method may imply a certain recruitment bias, the involvement of densely populated urban areas in four countries entails a level of randomness that allows for a reasonable degree of external validity (at least when it comes to the same targeted regions).

There did appear to be cultural and societal differences that may help explain the observed variations across the four countries. Indeed, as shown in Table 1, the U.S., England, India, and Poland, showed differences in development, economic conditions, and violence-related metrics that likely shaped the predictive outcomes. In this context, the U.S., for example, reported the highest GDP per capita ($85,373) and gun ownership (120 guns per 100 people), as well as a disproportionate share of global gun deaths (14.85%). These factors, perhaps uncoincidentally, align with its elevated homicide rate (4.96 per 100,000) and general violence rate (3.96 per 100,000). In contrast, the United Kingdom exhibited a higher Human Development Index (HDI) score (.940), but much lower rates of gun deaths, violence, and homicide, suggesting a societal context with stricter gun control measures and potentially stronger institutional mechanisms to mitigate violence.

Interestingly, India presented a markedly different profile, with the lowest HDI (.644) and GDP per capita ($10,123), coupled with the highest stabbing mortality rate (.64) among the countries reported. This suggests that resource constraints and differing cultural or societal norms regarding weapon use might play a role in the patterns of violence observed. Meanwhile, Poland occupied a middle ground, with an HDI (.881) and GDP per capita ($49,060) that reflected its transitional economic status, and relatively low rates of gun ownership (2.50 per 100 people) and homicide (.73 per 100,000), which might be attributed to effective violence prevention policies and cultural attitudes toward weapon use.

Thus, further research should involve more national regions and bigger samples with more internal variance, which would be particularly relevant to large and diverse countries, like the US or India, and which might even result in regionally specific models. Such research might go beyond the current cross-sectional design, include other weapons (such as the baseball bat that is popular in Poland), focus exclusively on violent offenders and include female participants as exploring the sex difference within the culture and across cultures in acceptance for weapons carrying could be interesting in theoretical and practical terms.

Despite assurances of anonymity and confidentiality, the paper administration of the survey in the Indian and partial Polish sample may have resulted in some more ‘socially desirable or acceptable’ responses. The significance of the knife (all 4 samples) and gun (US, English and Polish samples) suggest that the survey mode did not play a major role, and apparently neither did the difference in recruitment via Facebook and on the campus although more caution is advisable when comparing the four samples, generalising from them and drawing implications.

Despite considerable differences between the explored cultures, the proposed Weapon Tolerance Model showed a pattern of (mostly) overlapping predictors and some factors that appear to be culture-specific (such as apparently higher weapon tolerance by Indian men). Further research might incorporate more of such specific factors to build even more elaborate and sensitive models structured around other types of violence. From a theoretical standpoint, this means that the overlap between theories that pertain to weapon-carrying in general [e.g., 54] and those that focus on specific weapons [55] should be considered further.

The results might potentially inform interventions aimed at reducing the male acceptance of guns and knives, e.g., through challenging aggressive masculinity in educational settings and popular culture. This might take the form of showing how counterproductive aggressive masculinity can be in comparison with more sensitive masculinities [56]. Such sensitive masculinities, however, would need to be presented as potentially more powerful face-saving tools than physical aggression, which could be promoted by role models who manage to stay calm in distress and under pressure [34,57].

Importantly, to be effective practical implications should endeavour to tap into culturally-specific values, which as in case of India’s Need for Respect, can play a large role. By identifying some cross-culturally important factors (Need for Respect and Aggressive Masculinity), this paper supports the development of campaigns that might have a wide-spread appeal across a range of cultural contexts. At the same time, it also appears that in weapon tolerance can be driven by different concerns in different cultures, which requires further investigation.

Overall, the presented results support the proposed Weapons Tolerance Model cross-culturally, but with some culture-specific idiosyncrasies. One potential reason for this might be the similarity of the generally monolithic cultural themes that tend to glamorise hegemonic themes of masculinity and violence. To make communities safer, policy makers need to question such themes and promote non-aggressive forms of manliness like emotional control or learned expertise.

## Supporting information

S1 File Weapons paper full data. (ZIP)
